# Field-Based Feasibility Assessment of Sorghum, Maize and Soybean for the Phytomanagement of Heavy Metal-Contaminated Mining Soils in a Living Lab Platform

**DOI:** 10.3390/toxics14070568

**Published:** 2026-06-28

**Authors:** Mădălina F. Ioniță, Emilia C. Dunca, Sorin M. Radu

**Affiliations:** 1 Department of Environmental Engineering and Geology, Faculty of Mining, University of Petroșani, 332006 Petroșani, Romania; madalina-flaviaionita@upet.ro; 2 Department of Mechanical, Industrial and Transport Engineering, University of Petroșani, 332006 Petroșani, Romania

**Keywords:** phytomanagement, post-mining soils, heavy metals, phytostabilisation, ecological rehabilitation, *Sorghum bicolor*, *Glycine max*, *Zea mays*, contaminated land, sustainable remediation

## Abstract

Heavy metal-contaminated post-mining soils remain persistent sources of ecological degradation and contaminant dispersion. This study provides a quantitative field-based assessment of sorghum (*Sorghum bicolor*), maize (*Zea mays*) and soybean (*Glycine max*) cultivated on heavy metal-affected mining soil from the Jiu Valley, Romania, within a Living Lab platform. Soil properties, pseudo-total metal concentrations, multi-year biomass production, growth indicators, vegetation cover and aboveground plant metal concentrations were evaluated. The soils showed slightly acidic to near-neutral pH, low organic matter and multi-metal contamination, with Cr, Cu, Ni, Zn and Pb ranging from 82 to 146, 51 to 92, 41 to 79, 156 to 287 and 64 to 121 mg kg^−1^, respectively. Total fresh biomass increased from 85 kg in the first cultivation year to 487 kg in the third cultivation year, with sorghum showing the highest final production (230 kg) and vegetation cover (55–86%). Aboveground Cr, Cu, Ni, Zn and Pb concentrations measured at species-specific levels of 6.21–8.06, 8.77–10.64, 7.48–12.92, 38.01–47.11 and 4.32–6.46 mg kg^−1^ dry weight, respectively. Sorghum showed the highest preliminary phytomanagement suitability, mainly through stronger vegetation cover formation, higher fresh biomass production and lower visible stress under the investigated field conditions. Maize showed intermediate feasibility, whereas soybean appeared more sensitive to the degraded substrate. Biomass reuse should be considered only under controlled non-food pathways, such as pyrolysis or anaerobic digestion, and should only be considered after a dedicated assessment of dry biomass, conversion residues and metal fate.

## 1. Introduction

Post-mining landscapes affected by heavy metal contamination are among the most environmentally vulnerable anthropogenic ecosystems. Mining residues, waste rock deposits, flotation tailings and abandoned industrial substrates frequently exhibit altered physicochemical properties, low biological activity, poor structural stability and elevated concentrations of potentially toxic elements. In many former mining regions, these degraded lands remain insufficiently rehabilitated decades after mine closure, generating persistent ecological and environmental risks associated with metal mobility, wind erosion, dust generation and surface runoff [1,2,3,4].

The ecological rehabilitation of contaminated mining land remains particularly challenging because conventional remediation technologies are often associated with high implementation costs, major landscape disturbance and limited applicability at large spatial scales. Excavation, soil replacement and physicochemical treatments may be technically effective under specific conditions; however, their long-term sustainability is frequently constrained in extensive post-mining environments characterised by heterogeneous contamination, limited accessibility and reduced economic viability. Consequently, low-impact, scalable and ecologically compatible remediation strategies are increasingly needed for the long-term management of degraded mining areas [5,6,7,8].

In recent years, phytomanagement has emerged as a realistic and sustainable approach for contaminated land rehabilitation. Unlike remediation strategies focused exclusively on pollutant removal, phytomanagement aims to establish stable and functional vegetation systems capable of reducing environmental risks while also supporting ecological recovery and potential socioeconomic benefits [9,10]. This approach includes phytostabilisation, assisted revegetation, erosion control, improvement of soil structure and the controlled utilisation of biomass generated on contaminated substrates. Therefore, phytomanagement should be understood primarily as a risk-reduction and ecological-stabilisation strategy rather than as a complete decontamination technique [9,10,11,12].

The success of phytomanagement strategies depends strongly on the selection of plant species capable of tolerating environmental stress associated with metal contamination, low nutrient availability, reduced organic matter content and degraded substrate conditions [1,5]. Species characterised by rapid establishment, relatively high biomass production, extensive root development and adaptability to marginal soils may contribute to the progressive stabilisation of post-mining landscapes. In this context, crops such as sorghum (*Sorghum bicolor*), soybean (*Glycine max*) and maize (*Zea mays*) have attracted increasing attention due to their physiological adaptability, biomass potential and possible integration into sustainable land management systems [1,5,13,14].

Sorghum is widely recognised for its tolerance to drought stress, extensive root system and capacity to grow under relatively poor soil conditions [5,15]. Maize is characterised by high biomass productivity and broad agronomic adaptability, including under contaminated or marginal soil conditions [3,16]. Soybean may provide additional ecological value through biological nitrogen fixation and contribution to soil biological functionality, although its performance can be affected by heavy metal stress and degraded substrate conditions [17,18]. However, these species may respond differently to mining-related stress, depending on substrate quality, metal occurrence, water availability and plant-specific tolerance mechanisms. Their comparative performance under real post-mining field conditions remains insufficiently documented, particularly in Eastern European mining regions [1,5,13].

Despite the increasing interest in phytomanagement of contaminated soils, field-based evaluations under real post-mining conditions remain limited. Most existing studies have focused on controlled pot experiments, single-species phytoremediation trials or short-term assessments of plant tolerance, while comparative Living Lab approaches integrating crop adaptability, vegetation establishment, soil stabilisation potential and cautious biomass reuse are still insufficiently explored. This knowledge gap is particularly relevant for former coal mining regions, where large degraded surfaces require remediation strategies that are technically feasible, economically realistic and compatible with local ecological and socioeconomic conditions [5,19,20,21].

The Jiu Valley, Romania, represents one of the most important historical coal mining regions in the country and includes numerous degraded mining areas affected by waste storage, industrial residues and long-term land disturbance. Following mining decline and progressive mine closure, large surfaces remain environmentally degraded and require sustainable rehabilitation approaches adapted to local post-mining landscapes. In this regional context, field-based pilot platforms can provide useful evidence regarding the feasibility of vegetation-based remediation strategies under real environmental conditions [22].

From a toxicological and environmental risk perspective, phytomanagement of metal-contaminated mining soils should not be evaluated only through contaminant removal. In many post-mining environments, the main short- and medium-term objective is to reduce exposure pathways by limiting bare soil surfaces, wind-driven dust generation, surface runoff and particle-bound contaminant redistribution. Therefore, vegetation establishment and surface stabilisation may represent relevant risk-reduction endpoints, particularly where complete soil removal or intensive physicochemical remediation is technically or economically unrealistic. In this context, field-based assessments are needed to identify plant species capable of tolerating multi-metal stress while contributing to soil cover formation and long-term ecological stabilisation [1,2,4,8].

Therefore, this study aimed to assess the preliminary field-based feasibility of sorghum, soybean and maize as candidate crops for the phytomanagement of heavy metal-contaminated mining soils from the Jiu Valley, Romania. The working hypothesis was that the three crops would differ in their capacity to establish vegetation cover, produce aboveground biomass and tolerate degraded substrate conditions, with sorghum expected to show higher stabilisation-oriented performance than maize and soybean. The study addressed three research questions: (i) which crop showed the strongest establishment and vegetation cover under mining-affected field conditions; (ii) how did aboveground biomass, plant height and plant density change across the monitored cultivation years; and (iii) whether aboveground plant metal concentrations and simple accumulation indicators support cautious interpretation of controlled non-food biomass management. By using a pilot Living Lab platform, this research supports the development of practical, nature-based remediation approaches, while recognising that complete phytostabilisation or contaminant removal cannot be demonstrated without additional root, bioavailability and post-cultivation soil assessments.

## 2. Materials and Methods

### 2.1. Study Area

The experimental study was conducted within a pilot Living Lab platform established on a mining-affected site located in the Jiu Valley, Romania. The region has a long history of coal extraction and associated industrial activities, which have generated extensive areas affected by waste deposits, tailings accumulation and progressive land degradation. The investigated site is representative of post-mining substrates characterised by disturbed soil structure, heterogeneous granulometry, limited vegetation cover and residual heavy metal contamination [8,22].

The regional climate is temperate continental, with seasonal thermal variability characteristic of mountain depression environments. For the Living Lab platform, the growing season was considered to extend from April to September, corresponding to the main period of crop establishment, vegetative development and aboveground biomass formation. For the 2024 growing season, representative local climatic data for the Petroșani area indicated a mean air temperature of approximately 18.6 °C and total precipitation of approximately 362 mm. These conditions suggest a moderate seasonal water constraint during the active vegetation period and are relevant for interpreting crop establishment, visible stress symptoms and differences in biomass production among the tested species. Because complete plot-scale meteorological records were not available for all three cultivation years, climatic information was used only to contextualise field performance and was not included as an explanatory variable in the statistical analysis.

Prior to the establishment of the experimental platform, the investigated land exhibited sparse spontaneous vegetation and reduced ecological functionality, including poor surface stability and limited soil cover. These characteristics made the site suitable for evaluating vegetation-based rehabilitation strategies under real post-mining field conditions.

**Figure 1:**
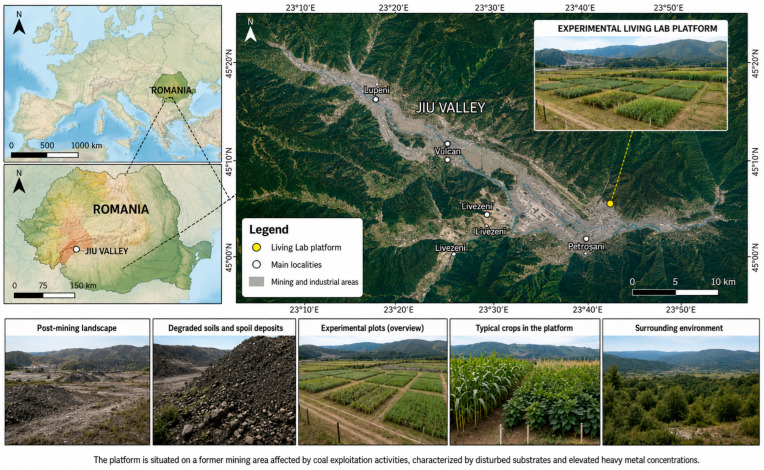
Location of the experimental Living Lab platform in the Jiu Valley, Romania, and representative field conditions of the post-mining area. The yellow point indicates the Living Lab platform, white circles indicate the main localities, and grey shading indicates mining and industrial areas Created with Canva (https://www.canva.com/, accessed on 27 April 2026).

The Living Lab platform was designed as a pilot field-based system for assessing the feasibility of phytomanagement as a nature-based remediation strategy focused on vegetation establishment, surface stabilisation, potential erosion risk and ecological rehabilitation of contaminated mining soils [5,9,14,19,20]. The location and regional context of the experimental Living Lab platform are shown in Figure 1.

### 2.2. Experimental Living Lab Design

The cultivated Living Lab experimental platform covered approximately 5000 m^2^ and included nine cultivation plots distributed among three crop species: sorghum (*Sorghum bicolor*), soybean (*Glycine max*) and maize (*Zea mays*). Each crop species was cultivated in three individual plots, coded as S1–S3 for sorghum, SO1–SO3 for soybean and M1–M3 for maize. Each cultivation plot measured 10 × 10 m (100 m^2^), while a central 8 × 8 m assessment area was used within each plot for standardised vegetation and biomass evaluation in order to reduce edge effects. The plots were treated as operational field replicates for the comparative assessment of crop establishment, vegetation development, biomass production and phytomanagement suitability.

The experimental layout followed an operational Living Lab arrangement rather than a fully randomised agronomic block design. The plots were organised systematically within the available field surface to allow access, monitoring and management under real post-mining conditions. Therefore, the three plots assigned to each species were considered operational spatial replicates for exploratory comparison, not fully independent experimental replicates. This design choice reflects the applied character of the platform, but it also limits the strength of inferential statistical conclusions and was considered when interpreting the results.

The field assessment was conducted over three consecutive cultivation years. In the manuscript, Year I, Year II and Year III refer to the first, second and third cultivation years of the Living Lab experiment, respectively. The three cultivation years correspond to 2023, 2024 and 2025. This notation was used to maintain consistency across biomass, growth and vegetation monitoring datasets.

Biomass production, plant height, plant density and vegetation cover were monitored across the three cultivation years, whereas aboveground plant tissue metal concentrations were determined at the final harvest of the third cultivation year. Representative field photographs were used only for visual documentation of crop establishment and vegetation development and were not used as quantitative evidence. Where photographs correspond to a specific growing season, this is indicated in the figure caption to avoid confusion between photographic documentation and the full three-year monitoring dataset.

The cultivated field units were established within the 5000 m^2^ Living Lab surface. For standardised monitoring and sampling, internal assessment areas were delimited within each plot, while the remaining surfaces functioned as cultivated area, access corridors and buffer zones. This design allowed repeated vegetation observations and biomass measurements under comparable post-mining field conditions while preserving the applied Living Lab character of the platform.

The platform was conceived as a pilot Living Lab rather than as a fully controlled agronomic trial. Therefore, the three plots assigned to each species were used for comparative interpretation and exploratory statistical screening, while acknowledging the inherent heterogeneity of the post-mining substrate. The results are interpreted as a field-based feasibility assessment for phytomanagement and not as a definitive agronomic productivity trial.

**Figure 2:**
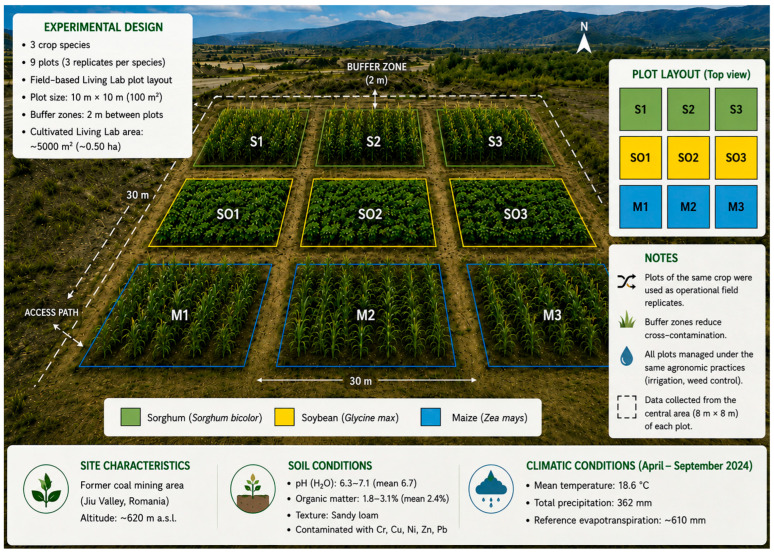
Schematic layout of the Living Lab experimental plots cultivated with sorghum, soybean and maize. Created with Canva (https://www.canva.com/, accessed on 27 April 2026).

The three species were cultivated under similar management conditions during the monitored vegetation cycles, without food- or feed-oriented production objectives. Sowing was performed manually in mid-May of each cultivation year, generally between 10 and 20 May, depending on soil moisture and field accessibility. Seeds were sown in rows spaced approximately 0.50 m apart. The target-sowing densities were approximately 25 seeds m^−2^ for sorghum, 20 seeds m^−2^ for maize and 30 seeds m^−2^ for soybean. These densities were selected to favour rapid vegetation cover formation rather than conventional food- or feed-oriented yield optimisation. Sowing depth was approximately 3–4 cm for sorghum and soybean and 4–5 cm for maize. Before sowing, the plots were subjected only to minimal surface preparation, consisting of manual levelling, removal of coarse surface residues and shallow loosening of the upper soil layer. No mineral or organic fertilisers, soil amendments, pesticides or herbicides were applied. Supplemental irrigation was applied uniformly to all plots only during crop establishment and during visibly dry periods, using the installed field irrigation system. Weed control was performed manually during the early growth stages to maintain plot accessibility and reduce competition. The focus was placed on ecological rehabilitation, surface stabilisation, quantitative crop performance and the preliminary evaluation of controlled non-food biomass potential [5,9,14]. The spatial arrangement of the nine cultivation plots and the main operational design features of the Living Lab platform are illustrated in Figure 2.

### 2.3. Soil Sampling and Physicochemical Characterisation

Soil sampling was performed before vegetation establishment in order to characterise the initial substrate conditions of the Living Lab platform. Composite soil samples were collected from the upper 0–20 cm soil horizon using a stainless-steel Edelman auger (Eijkelkamp Soil & Water, Giesbeek, The Netherlands), corresponding to the main rooting zone for early crop establishment and the soil layer most directly involved in surface stabilisation processes. For each experimental plot, multiple subsamples were collected from different positions within the plot and homogenised to obtain one representative composite sample per plot. In total, nine composite soil samples were obtained, corresponding to the nine cultivation plots [23].

The collected samples were transported to the laboratory in polyethylene bags, air-dried at room temperature, manually homogenised, gently disaggregated and prepared for laboratory analysis according to standard soil sample preparation procedures [23]. Soil pH was determined potentiometrically in aqueous suspension using a WTW Multi 3630 IDS multiparameter instrument equipped with a pH electrode (WTW/Xylem Analytics, Weilheim, Germany), calibrated with standard pH 4.00, 7.00 and 10.00 buffer solutions (WTW/Xylem Analytics, Weilheim, Germany) [24]. Organic matter content was estimated by loss on ignition using pre-dried soil material, after oven drying at 105 °C in a laboratory drying oven (Memmert GmbH + Co. KG, Schwabach, Germany) and ignition at 550 °C in a laboratory muffle furnace (Nabertherm GmbH, Lilienthal, Germany) available in the laboratory facilities of the University of Petroșani, Petroșani, Romania [25]. The investigated metals were chromium (Cr), copper (Cu), nickel (Ni), zinc (Zn) and lead (Pb), selected based on their environmental relevance for mining-affected soils and their potential contribution to ecological risk [22,26].

### 2.4. Heavy Metal Analysis and Quality Assurance

Heavy metal occurrence was evaluated after acid digestion of the prepared soil samples. The digestion procedure, performed using a microwave digestion system (Milestone ETHOS UP, Milestone Srl, Sorisole, Italy), followed microwave-assisted acid digestion, adapted from U.S. EPA Method 3051A, commonly applied for the determination of pseudo-total metal concentrations in soils, sediments, sludges and related solid matrices [27]. Analytical-grade reagents, including nitric acid (HNO_3_, 65%; Merck KGaA, Darmstadt, Germany) and hydrochloric acid (HCl, 37%; Merck KGaA, Darmstadt, Germany), were used throughout the digestion procedure. Accordingly, the obtained values were interpreted as indicators of the overall contamination status of the investigated substrate rather than as direct measures of metal bioavailability.

After digestion, the extracts were filtered through Whatman Grade 42 ashless filter papers (Cytiva, Marlborough, MA, USA), diluted to a known volume with deionised water obtained using a Milli-Q water purification system (Merck Millipore, Burlington, MA, USA) and analysed for Cr, Cu, Ni, Zn and Pb using inductively coupled plasma optical emission spectrometry, ICP-OES (PerkinElmer Avio 200/500 series, PerkinElmer, Waltham, MA, USA), following the analytical principles of U.S. EPA Method 6010D [28]. Metal concentrations were expressed as mg kg^−1^ dry weight [28].

Analytical quality assurance and quality control included external calibration, calibration verification, procedural blanks and replicate measurements. Calibration was performed using Merck Certipur multi-element calibration standards (Merck KGaA, Darmstadt, Germany), selected according to the analysed elements and expected concentration ranges. Calibration verification standards were analysed during the analytical sequence to check instrumental stability. Procedural blanks were processed through the same preparation, digestion and analytical steps as the soil samples in order to identify potential contamination during sample handling and digestion. Replicate measurements were used to evaluate analytical precision and to verify the consistency of the obtained concentrations.

The QA/QC procedure ensured that the analytical results were suitable for baseline contamination characterisation of the Living Lab substrate and for supporting the exploratory interpretation of crop performance under mining-affected field conditions. However, matrix-matched certified reference material results, element-specific recovery values and complete method detection and quantification limits were not available for all soil and plant tissue analyses. Therefore, the metal concentration data were interpreted as screening-level analytical evidence rather than as regulatory-grade certification data. Because the digestion procedure provides pseudo-total concentrations, the results were not interpreted as direct indicators of metal bioavailability or plant-available fractions.

### 2.5. Vegetation Monitoring and Biomass Assessment

Vegetation development was monitored periodically over three consecutive cultivation years, from crop establishment to final harvest in each growing season. Monitoring focused on indicators directly relevant to phytomanagement under degraded post-mining conditions, including emergence and establishment success, plant height, plant density, vegetation cover, canopy development, visible stress symptoms and aboveground biomass formation [5,9,19,20]. In the results, the three monitoring years are reported as Year I, Year II and Year III, corresponding to the first, second and third cultivation cycles of the Living Lab platform. The three cultivation years correspond to 2023, 2024 and 2025.

The main field evaluations were carried out at emergence, at approximately 4, 8 and 12 weeks after emergence, and at maturity/harvest. Plant height was measured using a graduated field measuring rod or measuring tape (Stanley Black & Decker, New Britain, CT, USA), while plant density was expressed as plants m^−2^. Vegetation cover was estimated as the percentage of soil surface covered by vegetation using 1 m^2^ field quadrat frames custom-made from PVC and prepared at the University of Petroșani, Petroșani, Romania, positioned in representative areas of each plot; the final value was expressed as the mean percentage cover recorded for the assessed surfaces. For each plot, vegetation cover was estimated using five 1 m^2^ quadrats positioned within the central 8 × 8 m assessment area of the plot. The quadrats were arranged systematically in a fixed five-point pattern, consisting of one central quadrat and four quadrats placed towards the internal corners of the assessment area. This arrangement was selected to avoid margin effects, access corridors and disturbed plot edges, while maintaining comparable assessment conditions across all plots. The quadrat observations were used only for estimating vegetation cover and visible stress, whereas fresh biomass was recorded separately according to the harvested area specified for the biomass assessment. The same quadrat-positioning scheme was maintained during the monitoring campaigns at approximately 4, 8 and 12 weeks after emergence and at maturity/harvest. The final vegetation cover value for each plot was calculated as the mean percentage cover recorded from the five quadrat-level observations.

Visible stress symptoms were assessed in the same central assessment area by two field observers, considering chlorosis, uneven emergence, reduced vigour, low plant density and growth reduction. Stress was classified using a four-level qualitative scale: absent (0), low (1), moderate (2) and severe (3). Low stress corresponded to slight or localised symptoms, moderate stress to clearly visible symptoms affecting plant vigour or uniformity, and severe stress to extensive symptoms associated with poor establishment or strong growth reduction. When observer assessments differed, the plot was jointly re-evaluated and the final category was assigned by consensus as the dominant plot-level stress class.

Fresh aboveground biomass was determined by weighing the harvested aerial material for each species and, where plot-level data were available, for each experimental plot, using a KERN field/laboratory balance (KERN & Sohn GmbH, Balingen, Germany). Because a complete dry-mass dataset was not available for all monitoring years, fresh biomass was used as the main quantitative biomass endpoint. Drying-bed observations were used only as supporting information and were not used for interannual statistical comparison.

Two biomass reporting scales were distinguished. Multi-year biomass values were reported at the species level as total fresh aboveground biomass harvested from the cultivated Living Lab plots during each cultivation year. In contrast, plot-level biomass values were used for standardised plot-level comparison in the last monitoring year. Therefore, these values were not interpreted as the same biomass accounting scale. To improve comparability, values were also expressed per unit area where the harvested area was known.

### 2.6. Plant Tissue Sampling and Heavy Metal Determination

Aboveground plant material was sampled at harvest for the determination of metal concentrations in plant tissues. The analysed material consisted of the total aerial biomass, represented by leaves and stems, and was processed as species-level composite samples for sorghum, maize and soybean. Root tissues were not analysed in this stage of the study. Chlorophyll content, ROS levels, programmed cell death markers, soil oxygen status and root tissue metal concentrations were not determined in the present field assessment; these parameters were therefore considered as limitations and future research needs.

Plant samples were manually cleaned to remove coarse impurities, rapidly washed with tap water followed by distilled/deionised water, drained on filter paper, dried in a laboratory drying oven (Memmert GmbH + Co. KG, Schwabach, Germany), ground using a laboratory plant mill/grinder (Retsch GmbH, Haan, Germany) and homogenised prior to digestion. Sample weighing was performed using an analytical balance (Mettler Toledo, Greifensee, Switzerland). The dried plant material was subjected to acid digestion using an oxidising digestion protocol based on hydrogen peroxide, H_2_O_2_, analytical grade (Merck KGaA, Darmstadt, Germany). The resulting solutions were analysed by inductively coupled plasma mass spectrometry, ICP-MS (PerkinElmer NexION 2000, PerkinElmer, Waltham, MA, USA), using Merck Certipur multi-element calibration standards (Merck KGaA, Darmstadt, Germany). Although the analytical protocol allowed multi-element screening, the present manuscript reports and interprets only Cr, Cu, Ni, Zn and Pb, because these elements were common to both the soil and plant datasets and were directly relevant to the phytomanagement assessment of the investigated mining-affected substrate. Concentrations were expressed as mg kg^−1^ dry weight.

### 2.7. Comparative Phytomanagement Suitability Assessment

The phytomanagement suitability of sorghum, maize and soybean was evaluated using a semi-quantitative field scoring framework designed for preliminary crop prioritisation under post-mining conditions. The assessment included five criteria directly relevant to risk-reduction-oriented phytomanagement: vegetation establishment, stress tolerance, biomass stability, contribution to ecological stabilisation and potential suitability for controlled non-food biomass use [5,9,19,20].

Potential suitability for controlled non-food biomass use was scored conservatively by considering both biomass production and aboveground plant metal concentrations. Because root accumulation, ash composition and contaminant fate during biomass conversion were not determined, this criterion was interpreted as a preliminary non-food management indicator rather than as proof of biomass safety [10,12,29,30].

Each criterion was scored from 1 to 5, where 1 indicated very low suitability and 5 indicated very high suitability. To reduce subjectivity, scores were assigned using predefined descriptors, as presented in Table 1. The overall phytomanagement suitability score was calculated as the sum of the five criterion scores, according to Equation (1):

Suitability score = S_1_ + S_2_ + S_3_ + S_4_ + S_5_ (1)

where S_1_–S_5_ represent the scores assigned to vegetation establishment, stress tolerance, biomass stability, contribution to ecological stabilisation and potential controlled non-food biomass use, respectively. The maximum possible score was 25.

The five criteria were assigned equal weight because the aim was to provide a simple and transparent screening tool for preliminary crop prioritisation, rather than to develop a validated multi-criteria decision model. The selected criteria were chosen to represent complementary but partly related dimensions of early-stage phytomanagement feasibility: establishment capacity, visible stress response, biomass development, surface cover contribution and cautious non-food biomass management. Because some of these dimensions are not fully independent, particularly vegetation establishment, biomass stability and contribution to ecological stabilisation, the resulting score may partially reflect overlapping field responses. Therefore, the score was used only for comparative screening within this pilot Living Lab platform and was not interpreted as an independently validated suitability index.

The scoring framework was used as a preliminary decision-support tool for comparing crop performance under real field conditions. It was not intended as a regulatory remediation index or as a toxicological risk index. Instead, it was designed to support the identification of candidate crops suitable for further phytomanagement trials by integrating field observations related to establishment capacity, stress response, vegetation development, surface stabilisation potential and cautious biomass reuse perspectives.

Given the pilot nature of the Living Lab platform, the limited number of field replicates and the descriptive character of the suitability framework, no inferential statistical testing was applied to the suitability scores. The results were interpreted descriptively and comparatively, with emphasis on crop prioritisation for remediation-oriented phytomanagement rather than on definitive statistical ranking.

### 2.8. Data Interpretation and Study Limitations

The results were interpreted in relation to the initial contamination status of the soil, crop establishment capacity, vegetation development and the potential contribution of each species to ecological stabilisation. The study focused on the feasibility of establishing tolerant biomass crops on mining-affected soils under real field conditions, rather than on complete contaminant removal or quantitative remediation efficiency.

Because the present assessment did not include detailed bioavailable or plant-available metal fractions, rhizosphere metal speciation, root metal accumulation, ash composition or contaminant fate during biomass conversion, the potential reuse of biomass generated on contaminated land was considered only as a prospective controlled non-food option. Aboveground plant metal concentrations were used as a first screening indicator of biomass contamination, but further analyses of roots, conversion residues and reuse scenarios are required before practical biomass valorisation pathways can be recommended [10,12,29,30].

Accordingly, all results are interpreted within the limits of a pilot field feasibility assessment. The study does not aim to quantify complete metal removal or long-term remediation efficiency, but to identify crop species capable of supporting vegetation establishment, biomass production, surface stabilisation and controlled non-food biomass perspectives under real post-mining conditions.

### 2.9. Statistical Analysis

The quantitative dataset was analysed using descriptive and exploratory statistics suitable for a pilot Living Lab design. For biomass, plant height, plant density and vegetation cover, mean values, standard deviations, ranges and percentage changes between monitoring years were calculated. Because the number of operational field replicates was limited (*n* = 3 plots per species) and the study was designed as a feasibility assessment rather than as a fully controlled agronomic experiment, non-parametric Kruskal–Wallis tests were used for exploratory comparisons among species where plot-level data were available. Spearman’s rank correlation was used to evaluate the association between fresh biomass and vegetation cover. Descriptive statistics, percentage changes, exploratory non-parametric comparisons and correlation analyses were performed using Microsoft Excel for Microsoft 365, Version 2505, 64-bit (Microsoft Corporation, Redmond, WA, USA). Statistical results were interpreted as screening evidence supporting crop prioritisation, not as definitive agronomic or toxicological rankings.

## 3. Results

### 3.1. Physicochemical Characteristics and Contamination Status of the Investigated Soils

The investigated soils displayed characteristics typical of mining-affected substrates, including heterogeneous structure, reduced organic matter content and detectable multi-metal contamination. Soil pH ranged from slightly acidic to near-neutral conditions, with values between 6.3 and 7.1 and a mean value of 6.7. These pH conditions may influence metal mobility and plant establishment, particularly in degraded substrates with limited buffering capacity and low biological activity [2,8,22].

Organic matter content ranged from 1.8 to 3.1%, with a mean value of 2.4%, indicating reduced organic enrichment compared with undisturbed productive soils. This limited organic matter content reflects the degraded status of the substrate and may contribute to lower nutrient availability, reduced microbial activity and constrained vegetation development [25].

The analysed soils contained pseudo-total concentrations of Cr, Cu, Ni, Zn and Pb, confirming the mining-related contamination context of the Living Lab platform. Among the investigated elements, Zn showed the highest mean concentration and the widest range, varying from 156 to 287 mg kg^−1^, while Cr ranged from 82 to 146 mg kg^−1^ and Pb from 64 to 121 mg kg^−1^. The occurrence of multiple metals indicates that the investigated substrate represents a relevant field context for testing vegetation-based remediation strategies [1,22].

The initial soil conditions across the nine composite samples collected before vegetation establishment are summarised in Table 2 using mean values and observed ranges.

To provide a national regulatory context, the measured pseudo-total metal concentrations were compared with the Romanian soil pollution assessment framework established by Order No. 756/1997 [26]. This comparison was used only to contextualise the contamination status of the Living Lab substrate and not to infer metal bioavailability or remediation efficiency. The analysed soils showed concentrations above the normal values for all investigated metals. Cr exceeded the alert threshold for sensitive land use in part of the samples, while remaining below the alert threshold for less sensitive land use. Cu exceeded the normal value but remained below the alert and intervention thresholds for both land-use categories. Ni was generally below the sensitive-use alert threshold, although the upper range slightly exceeded this value. Zn exceeded the normal value but remained below the alert thresholds for both land-use categories. Pb exceeded the alert threshold for sensitive land use, and the upper range locally exceeded the intervention threshold for sensitive land use, while remaining below the alert and intervention thresholds for less sensitive land use. The regulatory interpretation of the measured pseudo-total metal concentrations in relation to Romanian threshold values is summarised in Table 3 [26].

Overall, the soil dataset confirms that the Living Lab platform was established on a degraded mining-affected substrate with low organic matter content and multi-metal enrichment. The comparison with Romanian regulatory thresholds further supports the relevance of the site for testing phytomanagement under field conditions, particularly as a risk-reduction approach aimed at vegetation establishment, surface stabilisation and limitation of contaminant dispersion [2,4,9].

However, these soil data should be interpreted as a baseline contamination characterisation rather than as a complete agronomic diagnosis of the substrate, because additional parameters controlling plant growth, such as texture, nutrient status, salinity, moisture regime and compaction, were not determined in this stage of the study [1,13].

### 3.2. Establishment and Vegetation Development Under Mining-Affected Conditions

The three investigated crop species showed different responses to the degraded mining substrate during the monitoring period. These differences were reflected not only in qualitative field observations, but also in measurable growth indicators, including plant height, plant density, vegetation cover and fresh aboveground biomass.

Across the monitored years, sorghum showed the strongest growth response. Mean plant height increased from 75 cm in Year I to 130 cm in Year III, while plant density increased from 14 to 20 plants m^−2^. Maize also improved over time, with plant height increasing from 60 to 125 cm and density from 16.5 to 19 plants m^−2^. Soybean remained the most sensitive crop, with substantially lower plant height values, increasing only from 8 to 12 cm, although plant density improved from 11 to 17 plants m^−2^.

Expressed as relative changes between Year I and Year III, plant height increased by approximately 73.3% for sorghum, 108.3% for maize and 50.0% for soybean. Plant density increased by approximately 42.9% for sorghum, 15.2% for maize and 54.5% for soybean. These percentage changes provide additional quantitative support for the observed interannual improvement in crop establishment and growth, while the absolute values still indicate stronger overall field performance for sorghum and maize compared with soybean.

Plot-level vegetation cover confirmed the same performance gradient. Sorghum provided the highest surface cover, ranging from 55% in S1 to 86% in S3, followed by soybean (30–70%) and maize (25–60%). Stronger cover formation observed for sorghum is directly relevant to preliminary phytostabilisation potential, because rapid and persistent vegetation cover may reduce exposed soil surfaces and may limit wind erosion, dust dispersion and runoff-related contaminant redistribution [2,4,8].

*Sorghum bicolor* showed the most favourable field response among the investigated species, combining high vegetation density, increasing plant height, strong surface cover and limited visible stress symptoms. *Zea mays* displayed intermediate to good performance, with substantial height and biomass increases but less uniform cover. *Glycine max* showed weaker field performance, particularly in relation to plant height and early visible stress, suggesting higher sensitivity to the combined effects of substrate degradation, low organic matter and metal-related stress.

Overall, the quantitative field indicators support the following adaptability gradient under the tested mining-affected soil conditions: sorghum > maize > soybean. This ranking indicates that sorghum is the most suitable candidate for early-stage phytomanagement, while maize can be considered a complementary biomass crop and soybean may require substrate improvement measures before wider application on degraded mining soils [15,16].

The qualitative field assessment is presented in Table 4, while the quantitative biomass, growth and vegetation cover indicators are detailed in Table 5 and Table 6. Plant tissue metal concentrations are reported separately in Table 7.

Because the platform had an applied Living Lab design, these quantitative values should be interpreted as operational field indicators rather than conventional agronomic yield parameters.

**Figure 3:**
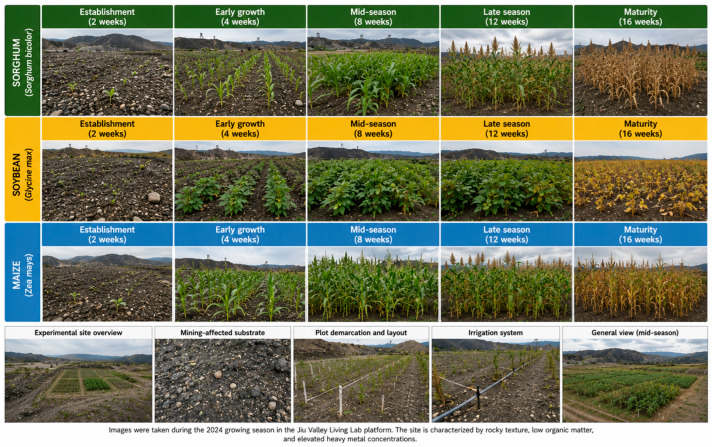
Representative field images documenting crop establishment and vegetation development of sorghum, soybean and maize during the 2024 growing season in the Jiu Valley Living Lab platform. The photographs illustrate the main visual growth stages from establishment to maturity and are used only as qualitative field documentation. They do not replace the three-year quantitative dataset on biomass, plant height, plant density and vegetation cover presented in Table 5 and Table 6.

Representative field images from the 2024 growing season are presented in Figure 3 to illustrate the visual development of the three crops from early establishment to maturity. These images are used as qualitative documentation of field conditions, whereas the quantitative interpretation of crop performance is based on the multi-year biomass, growth and vegetation cover data reported in Table 5 and Table 6.

### 3.3. Biomass Development and Implications for Surface Stabilisation

The biomass dataset showed a clear increase in total fresh aboveground biomass across the monitored years. Total fresh biomass increased from 85 kg in Year I to 263 kg in Year II and 487 kg in Year III, corresponding to an overall increase of approximately 473% between Year I and Year III. This trend indicates progressive improvement of vegetation establishment and biomass formation on the Living Lab platform [5,14].

Sorghum produced the highest amount of fresh biomass in all three monitoring years, increasing from 46 kg in Year I to 125 kg in Year II and 230 kg in Year III. Maize increased from 13 to 126 kg, while soybean increased from 26 to 131 kg. In Year III, sorghum accounted for approximately 47.2% of the total fresh biomass, confirming its role as the dominant biomass-producing crop under the investigated post-mining field conditions.

Descriptive statistics across the three monitoring years further support the stronger biomass performance of sorghum. Mean fresh biomass was 133.7 ± 92.3 kg for sorghum, compared with 72.3 ± 56.7 kg for maize and 72.3 ± 53.6 kg for soybean. At the plot level, sorghum also showed the highest mean fresh biomass (15.33 ± 8.86 kg), followed by soybean (5.67 ± 1.26 kg) and maize (4.33 ± 4.07 kg).

Exploratory non-parametric testing, applied cautiously because of the low number of operational spatial replicates, did not indicate statistically significant differences among species for plot-level fresh biomass at the conventional *p* < 0.05 threshold (Kruskal–Wallis H = 5.60, *p* = 0.061). However, the observed ranking suggested a biomass trend that requires confirmation in a larger, randomised and spatially replicated field experiment.

Spearman’s rank correlation indicated a strong positive association between plot-level fresh biomass and vegetation cover (ρ = 0.917, *p* = 0.0005). This result supports the interpretation that higher aboveground biomass formation was generally associated with stronger surface cover development across the Living Lab plots. However, because the analysis was based on a limited number of operational field replicates, the correlation should be interpreted as exploratory screening evidence rather than as definitive proof of surface stabilisation efficiency.

Taken together, the biomass and vegetation cover results indicate that stronger aboveground biomass formation was generally associated with higher surface cover, especially in the sorghum plots. This relationship should be interpreted as screening-level evidence of potential surface stabilisation rather than as direct proof of reduced erosion, dust dispersion or contaminant redistribution, because these processes were not directly measured in the present study [2,4].

### 3.4. Aboveground Plant Metal Concentrations

The analysis of aboveground biomass confirmed the occurrence of metals in plant tissues, but the observed concentrations remained species-specific and substantially lower than the pseudo-total concentrations measured in soil. Because only aerial biomass composites were analysed, the results should be interpreted as screening indicators of aboveground metal occurrence and not as complete metal uptake or phytoextraction efficiency [6,31].

Among the investigated elements, Zn showed the highest concentrations in plant tissues, ranging from 38.01 mg kg^−1^ dry weight in sorghum to 47.11 mg kg^−1^ dry weight in maize. Ni ranged from 7.48 mg kg^−1^ in soybean to 12.92 mg kg^−1^ in maize, while Cr ranged from 6.21 mg kg^−1^ in sorghum to 8.06 mg kg^−1^ in soybean. Pb concentrations were between 4.32 and 6.46 mg kg^−1^, and Cu ranged from 8.77 to 10.64 mg kg^−1^.

The plant tissue data support a cautious phytomanagement interpretation. Sorghum combined the highest biomass production with the lowest aboveground Cr concentration among the tested crops, while maize showed the highest aboveground Cu, Ni, Zn and Pb concentrations. Soybean presented the highest Cr concentration but the weakest growth response. Therefore, none of the tested crops should be considered suitable for food or feed use on contaminated mining soils, and any non-food utilisation must remain conditional upon residue-level metal assessment [12,29,30]. The species-level concentrations of the selected metals in aboveground biomass are presented in Table 7.

### 3.5. Screening-Level Accumulation Indicators in Aboveground Biomass

To provide an additional screening-level interpretation of metal transfer to aboveground biomass, an aboveground accumulation ratio (AR) was calculated for each species and metal using Equation (2):

(2) $$AR=\frac{C_{plant}}{C_{soil}}$$

where C_plant_ represents the metal concentration measured in aboveground plant biomass (mg kg^−1^ dry weight), and C_soil_ represents the mean pseudo-total concentration of the same metal in soil (mg kg^−1^ dry weight).

Because C_soil_ was based on pseudo-total soil concentrations rather than plant-available metal fractions, AR values were not interpreted as true bioaccumulation factors. Instead, they were used only as preliminary screening indicators of the relative transfer of metals from the contaminated substrate to aboveground biomass [6,7,31]. The calculated AR values for the three tested crops are presented in Table 8.

All calculated aboveground accumulation ratios were below 1, indicating that aboveground tissues contained substantially lower metal concentrations than the mean pseudo-total concentrations measured in soil. Maize showed the highest screening-level ratios for Cu, Ni, Zn and Pb, whereas soybean showed the highest ratio for Cr. These results should be interpreted cautiously because root tissues and plant-available metal fractions were not analysed. Therefore, the ratios do not allow distinction between phytoextraction and phytostabilisation, but they support a more transparent preliminary comparison of aboveground metal occurrence among the tested crops [6,7,31].

Total aboveground metal loads were not calculated because complete measured dry biomass data were not available, and plant tissue concentrations were obtained from species-level composite samples rather than from plot-level replicated samples. Since plant metal concentrations were expressed on a dry weight basis, calculating total metal loads from fresh biomass would have introduced additional uncertainty related to species-specific water content, harvest timing and moisture variability. Therefore, metal load estimation was considered a future analytical step requiring measured dry biomass, plot-level plant tissue data and root–shoot metal partitioning [12,29].

### 3.6. Semi-Quantitative Phytomanagement Suitability Assessment

The comparative suitability assessment indicated a higher preliminary phytomanagement potential of sorghum under the investigated field conditions. The scoring approach integrated field observations with quantitative indicators related to biomass production, growth performance, vegetation cover and aboveground plant metal concentrations.

Sorghum obtained the highest overall suitability due to its stable establishment, reduced visible stress, strong vegetation cover, highest biomass production and comparatively favourable aboveground metal profile. Maize showed moderate to high suitability, reflecting its strong biomass increase but lower cover uniformity and higher aboveground concentrations of several metals. Soybean obtained the lowest suitability score because of weaker growth, reduced plant height and greater sensitivity during early development. The overall suitability scores followed the order of *Sorghum bicolor* > *Zea mays* > *Glycine max*, with scores of 24/25, 19/25 and 14/25, respectively.

The scoring system should be interpreted as a preliminary decision-support tool rather than as a quantitative remediation index or an independently validated suitability model. It does not replace detailed measurements of bioavailable metal fractions, root–shoot metal partitioning, ash composition or long-term contaminant dynamics. Because the five criteria were equally weighted and partially related to one another, especially vegetation establishment, biomass stability and contribution to ecological stabilisation, the overall score may include some degree of overlap among field-performance indicators. Therefore, the score was used only to organise the observed field evidence and to support preliminary crop prioritisation within this pilot Living Lab platform [9,31].

The results are intended to support crop prioritisation for further phytomanagement trials, not to provide definitive remediation efficiency rankings. The resulting semi-quantitative phytomanagement suitability scores are summarised in Table 9.

To increase transparency in the semi-quantitative scoring approach, the field and quantitative evidence used to justify each assigned score is presented in Table 10. This table links the scores to the main visual observations, biomass results, vegetation cover indicators and aboveground plant metal data recorded during the field assessment. However, Table 10 should not be interpreted as an independent validation of the scoring system, because the same field observations and quantitative indicators informed the score allocation.

### 3.7. Implications for Phytomanagement-Oriented Rehabilitation

The results indicated that the tested crops may contribute differently to phytomanagement strategies for contaminated post-mining soils. Sorghum appears to be the most promising species for early vegetation establishment and surface stabilisation, especially where the primary objective is to reduce bare soil exposure and support ecological recovery. Maize may be suitable as a complementary biomass crop under moderate substrate stress, while soybean appears less appropriate as a primary stabilisation species under the investigated conditions [5,9].

**Figure 4:**
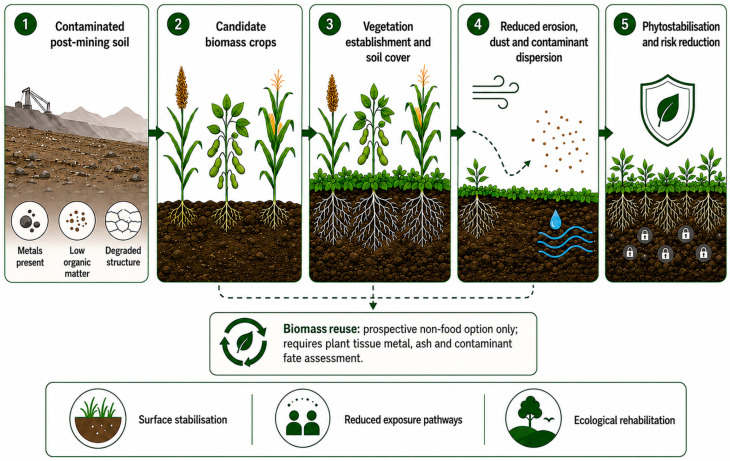
Conceptual workflow of field-based phytomanagement for co-contaminated post-mining soils. Created with Canva (https://www.canva.com/, accessed on 29 April 2026).

The comparative phytomanagement framework presented in Figure 4 summarises the main processes through which tolerant biomass crops may contribute to the rehabilitation of contaminated mining soils. These include vegetation establishment, soil surface protection, potential reduction of erosion susceptibility and dust dispersion risk, gradual improvement of ecological functionality and the prospective generation of biomass for controlled non-food uses [2,9].

The suitability gradient discussed in this section is supported by the field and quantitative evidence summarised in Table 10, but it should be interpreted within the exploratory scope of the Living Lab assessment. Sorghum obtained the highest score because it combined uniform establishment, low visible stress, stable canopy development, the highest multi-year biomass production and the strongest vegetation cover. Maize showed good but less uniform performance and higher aboveground concentrations of several metals, whereas soybean was more sensitive to the degraded substrate conditions. The score assigned to the controlled non-food biomass use criterion was therefore differentiated among species and interpreted conservatively, because only aboveground composite plant metal data were available, and root accumulation, ash composition and contaminant fate during biomass conversion were not assessed. Because the scoring framework was not independently validated and because some criteria partially overlap, the resulting ranking should be interpreted only as a preliminary field-based prioritisation under the specific conditions of this Living Lab platform, not as a definitive suitability classification.

The workflow illustrates the transition from contaminated substrate to vegetation establishment, soil cover development, potential reduction of erosion- and dust-related contaminant dispersion risk, and phytostabilisation-oriented risk reduction.

The framework supports the interpretation of tolerant biomass crops primarily as tools for vegetation establishment, surface stabilisation and remediation-oriented land management. Under contaminated soil conditions, biomass generation should be regarded as a secondary and conditional output, suitable only for carefully controlled non-food pathways after metal-fate assessment [10,12].

## 4. Discussion

### 4.1. Phytomanagement as a Remediation Strategy for Post-Mining Soils

The rehabilitation of mining-affected landscapes requires remediation strategies that are technically feasible, environmentally compatible and applicable over large degraded areas. In this context, phytomanagement represents a practical nature-based approach because it does not rely exclusively on complete contaminant removal, but on risk reduction, ecological stabilisation and progressive recovery of soil functionality. Recent reviews further emphasise that successful phytoremediation and phytomanagement strategies require the integration of plant tolerance, metal uptake or stabilisation behaviour, plant efficiency indicators, soil amendments and long-term monitoring under site-specific environmental conditions [1,2,5,9,13,31].

The results of the present study support the use of tolerant vegetation systems as an initial step in the rehabilitation of heavy metal-contaminated post-mining soils. Establishing plant cover on degraded substrates may reduce the exposure of bare soil surfaces, limit wind erosion and dust dispersion, and improve surface stability. These effects are particularly important in mining-affected environments, where poor soil structure, low organic matter content and heterogeneous contamination may maintain long-term ecological vulnerability [2,21,32,33].

The comparison with Romanian regulatory thresholds further reinforces the need for risk-reduction measures on the investigated substrate. The exceedance of normal values for all analysed metals and the local exceedance of sensitive-use alert or intervention thresholds for some elements indicate that the site represents a relevant field context for testing phytomanagement-oriented rehabilitation. Under such conditions, the establishment of stable vegetation cover can be considered a meaningful remediation endpoint, especially when the objective is to reduce contaminant dispersion rather than to achieve rapid decontamination.

Compared with intensive remediation technologies, such as excavation, soil replacement or physicochemical treatment, phytomanagement may offer a more realistic option for extensive post-mining areas where complete soil removal is economically and technically difficult. However, its effectiveness should be interpreted in relation to clearly defined objectives. In the present study, the primary remediation-oriented objective was not the rapid removal of metals from soil, but the identification of crop species capable of supporting phytostabilisation, vegetation establishment, surface protection and ecological rehabilitation under field conditions [3,4,5,9].

The Living Lab approach used in this study provides practical value because it evaluates crop behaviour under real post-mining conditions rather than under fully controlled experimental settings. This is relevant for the development of scalable remediation strategies, as field performance may differ substantially from greenhouse or pot-based responses due to substrate heterogeneity, climatic variability, water availability and local contamination patterns. Therefore, the present study contributes to the selection of candidate crops for risk-reduction-oriented phytomanagement in mining-affected landscapes [5,19,20].

### 4.2. Toxicological and Environmental Relevance of Phytomanagement

The toxicological relevance of phytomanagement in post-mining soils is related not only to the total concentration of metals in the substrate, but also to the probability of contaminant mobilisation, dispersion and exposure. In degraded mining areas with sparse vegetation cover, exposed soil particles may contribute to wind-driven dust generation, surface runoff and the redistribution of metal-bearing material towards adjacent soils, drainage pathways or human-used areas. Under such conditions, the establishment of stable vegetation cover can reduce relevant exposure pathways even when pseudo-total soil metal concentrations remain largely unchanged [1,2,9,13].

In the present study, the regulatory comparison with Romanian soil quality thresholds showed that the investigated substrate exceeded normal values for all analysed metals, while Cr, Ni and Pb locally exceeded sensitive-use alerts or intervention thresholds. These exceedances reinforce the relevance of the Living Lab platform as a field context for testing phytomanagement as a risk-reduction strategy rather than as a complete decontamination technology. From this perspective, the main remediation-oriented endpoint is the reduction of bare soil exposure, erosion susceptibility and particle-bound metal dispersion, especially during the early stages of ecological rehabilitation.

The observed crop performance gradient is therefore important from a risk-reduction perspective. Sorghum showed the most stable establishment, highest vegetation density and strongest contribution to soil cover formation, while maize provided an intermediate-to-high stabilisation potential. By contrast, soybean showed weaker establishment and more evident stress symptoms, suggesting a lower capacity to rapidly protect exposed mining-affected soil surfaces under the tested conditions. Consequently, sorghum and, to a lesser extent, maize may have greater potential to reduce erosion-related contaminant transport and ecological exposure under field conditions, but this effect requires confirmation through direct erosion, runoff or dust-dispersion measurements.

This interpretation is consistent with the phytostabilisation concept, where the remediation objective is not rapid metal extraction, but the limitation of contaminant mobility, dispersion and exposure through vegetation cover, root development and progressive improvement of surface stability. Therefore, the suitability scores obtained in this study should be interpreted primarily as indicators of phytomanagement and risk-reduction potential, rather than as indicators of contaminant removal efficiency or toxicological safety of the produced biomass [2,4,8].

### 4.3. Species-Specific Performance Under Mining-Affected Conditions

*Sorghum bicolor* showed the most favourable response under the investigated field conditions. Its stable establishment, high vegetation density and reduced visible stress symptoms were supported by the quantitative dataset. Fresh biomass increased from 46 kg in Year I to 230 kg in Year III, plant height increased from 75 to 130 cm, and vegetation cover reached 55–86% at the plot level. These values indicate that sorghum was able to establish a relatively dense and persistent aboveground cover on the investigated post-mining substrate. This response is consistent with recent studies that describe sorghum as a drought-adapted, high-biomass crop with potential for phytoremediation and phytomanagement of heavy metal-contaminated soils [5,14,15]. However, the biomass values reported in the present study are fresh aboveground biomass values and should not be directly compared with conventional dry-matter yields reported in agronomic productivity trials.

Compared with sorghum, *Zea mays* showed moderate to high suitability, combining satisfactory establishment with a substantial increase in fresh biomass from 13 kg in Year I to 126 kg in Year III. Plant height increased from 60 to 125 cm, indicating good growth capacity under the investigated conditions. Nevertheless, plot-level vegetation cover was lower and less uniform than in sorghum, ranging from 25% to 60%, which suggests a weaker contribution to continuous surface protection at plot scale. The aboveground plant tissue data also showed the highest Cu, Ni, Zn and Pb concentrations among the three crops. This is in line with studies indicating that maize can take up and translocate metals under contaminated soil conditions, which supports its phytoremediation relevance but also reinforces the need to restrict biomass use to non-food and carefully controlled pathways [3,12,16]. Therefore, maize may be useful as a complementary biomass crop in phytomanagement systems, but its harvested biomass requires dedicated metal-fate assessment before any valorisation pathway is considered.

*Glycine max* was comparatively more sensitive to the degraded mining substrate. Although fresh biomass increased from 26 kg in Year I to 131 kg in Year III, plant height remained low, ranging only from 8 to 12 cm, indicating constrained vertical development. Vegetation cover ranged from 30% to 70%, but this cover was associated with weaker growth vigour and more visible stress symptoms during early development. The response observed in soybean is consistent with recent literature showing that heavy metal stress can reduce soybean productivity, disturb physiological functioning and require microbial or amendment-assisted strategies to improve plant tolerance. As a leguminous crop, soybean may offer ecological benefits through biological nitrogen fixation and contribution to soil biological activity, but these benefits may be limited under mining-affected conditions when root development, symbiotic activity and nutrient availability are constrained [1].

The aboveground metal concentrations measured in the present study provide additional context for interpreting species performance. Zn showed the highest concentrations in plant tissues, ranging from 38.01 to 47.11 mg kg^−1^ dry weight, while Cr, Cu, Ni and Pb remained within lower concentration ranges of 6.21–8.06, 8.77–10.64, 7.48–12.92 and 4.32–6.46 mg kg^−1^ dry weight, respectively. In addition, all screening-level aboveground accumulation ratios were below 1, with the highest values observed for Zn in maize and soybean. These results indicate limited aboveground metal transfer relative to the mean pseudo-total soil concentrations, but they should not be interpreted as evidence of complete phytostabilisation or biomass safety because root tissues, plant-available metal fractions and conversion residues were not analysed. Overall, the comparison with recent phytomanagement and phytoremediation studies supports the interpretation that sorghum showed the strongest preliminary potential for surface cover formation and early-stage stabilisation of this specific post-mining substrate, maize showed complementary biomass potential with higher aboveground metal occurrence, and soybean may require assisted phytomanagement measures before wider field application [5,6,9,31].

### 4.4. Biomass Reuse: Opportunities and Toxicological Constraints

The production of biomass on contaminated mining soils represents both an opportunity and a potential environmental concern within phytomanagement systems. From a land management perspective, non-food biomass crops may support the productive reuse of degraded land and contribute to circular economy objectives, particularly when biomass is directed towards controlled industrial or bioenergy-oriented pathways, such as pyrolysis or anaerobic digestion. However, such reuse cannot be assumed to be environmentally safe without a dedicated assessment of contaminant transfer and residue management [10,11,12,30].

The aboveground plant tissue results obtained in this study provide a first indication of metal occurrence in the harvested biomass. The detected concentrations of Cr, Cu, Ni, Zn and Pb confirm that plant material produced on contaminated mining soils should not enter food or feed chains. Their potential role should therefore be limited to controlled non-food applications within remediation-oriented land management systems [10,12,29].

For energy-oriented valorisation, pyrolysis may be considered a potentially relevant pathway because it can convert biomass into biochar, condensable fractions and gases; however, metals may concentrate in char or ash residues and require strict characterisation before reuse or disposal. Anaerobic digestion may also be technically possible, but metals may accumulate in the digestate, which means that digestate reuse should be restricted to non-food, non-productive or mining-degraded land only after regulatory and ecotoxicological verification. The same precaution applies to any proposal to use resulting residues as biofertilisers on mining-origin degraded soils [12,29,30].

This distinction is important for the toxicological interpretation of phytomanagement systems. A crop may be suitable for vegetation establishment and surface stabilisation, but not automatically safe for biomass valorisation. Future implementation should therefore consider two separate evaluation levels: first, the ecological role of crops in phytostabilisation and risk reduction; second, the environmental safety of any biomass-reuse pathway, including metal distribution in plant tissues, ash, biochar, digestate and other conversion residues.

The present study does not fully validate biomass valorisation from contaminated mining soils. Instead, it identifies candidate crops that may generate sufficient vegetation cover and aboveground biomass to support phytostabilisation-oriented land management and provides a first screening of aboveground metal concentrations. Any future use of harvested biomass must remain conditional upon dedicated analyses of metal partitioning between roots and shoots, biomass quality, ash composition, digestate quality and contaminant behaviour during processing or conversion. Therefore, biomass reuse should be regarded as a secondary and controlled component of the phytomanagement strategy, not as an unrestricted utilisation pathway [10,12,29].

### 4.5. Study Limitations and Future Research Needs

This study should be interpreted as a field-based feasibility assessment rather than as a complete validation of long-term remediation efficiency. This positioning is important because early-stage phytomanagement trials are designed primarily to identify tolerant species capable of establishing vegetation cover under contaminated and structurally degraded substrate conditions, while long-term contaminant stabilisation and soil functionality improvement require extended monitoring.

Several limitations should therefore be acknowledged. First, the study evaluated pseudo-total metal concentrations in soil, but did not include a detailed assessment of bioavailable or plant-available metal fractions. Consequently, the measured concentrations provide a contamination context for interpreting crop performance, but they do not directly describe metal mobility or uptake potential. In addition, soil characterisation was limited to pH, organic matter and pseudo-total concentrations of Cr, Cu, Ni, Zn and Pb. Other soil and agronomic parameters that may influence plant establishment and biomass formation, including texture, electrical conductivity, available nitrogen, phosphorus and potassium, cation exchange capacity, bulk density, water-holding capacity, soil moisture, salinity and compaction, were not determined in the present phase of the Living Lab assessment. Consequently, the weaker growth response observed for soybean cannot be attributed exclusively to metal-related stress. It may also reflect the combined influence of nutrient limitations, soil structural constraints, water availability and substrate heterogeneity. Future monitoring should therefore include a broader soil characterisation before and after cultivation in order to separate contaminant-related effects from general edaphic constraints and to evaluate whether vegetation establishment leads to measurable improvement in soil quality over time. A further analytical limitation concerns the completeness of the QA/QC dataset. Although calibration verification, procedural blanks and replicate measurements were used to control instrumental stability, potential contamination and analytical repeatability, matrix-matched certified reference materials, full recovery data and element-specific method detection and quantification limits were not available for all analysed soil and plant matrices. This limits the analytical strength of the trace-metal dataset and supports the interpretation of the results as screening-level field evidence. Future analyses should include matrix-matched certified reference materials, recovery assessment, limits of detection and quantification, and replicate precision indicators for both soil and plant tissue samples. Another limitation is that plant tissue analysis was limited to aboveground composite biomass samples; root accumulation, root–shoot partitioning and species-specific metal transfer factors at plot scale were not determined. For this reason, the suitability of the tested crops should be interpreted primarily in relation to vegetation establishment, stress tolerance, biomass formation, surface stabilisation and preliminary aboveground metal screening, not as evidence of complete biomass safety or metal removal efficiency. In addition, total metal loads in aboveground biomass were not estimated in the present study because measured dry biomass was not available for all species and plant tissue concentrations were obtained from species-level composite samples. This limitation is particularly relevant because fresh biomass depends on species-specific water content and harvest conditions. Therefore, future assessments should combine measured dry biomass, plot-level plant tissue analyses and root–shoot partitioning in order to quantify metal loads and better distinguish between phytostabilisation-oriented and phytoextraction-oriented plant responses [1,6,31].

Physiological and rhizosphere-level stress indicators were also not determined in the present phase of the study. Chlorophyll content, photosynthetic performance, reactive oxygen species accumulation, programmed cell death markers and soil oxygen status were not measured. Consequently, visible stress symptoms observed in the field, such as chlorosis, reduced vigour and uneven growth, cannot be mechanistically linked to specific physiological responses or to oxygen-related constraints in the root zone. Future studies should therefore include chlorophyll-based indicators, oxidative stress markers, programmed cell death assessment, soil oxygen or redox-related measurements and detailed root tissue analysis in order to better explain plant tolerance mechanisms under heavy metal-contaminated post-mining field conditions [1,13].

A further limitation is related to the experimental design. Despite the inclusion of multi-year biomass and growth indicators, the Living Lab design involved a limited number of operational field replicates. The semi-quantitative suitability scoring system was designed as a practical decision-support tool for crop prioritisation under field conditions and should not be interpreted as a regulatory remediation index, a toxicological risk index or a definitive statistical ranking [19,20].

The absence of an unplanted control plot, a spontaneous vegetation reference area or an uncontaminated reference soil represents another limitation of the present Living Lab assessment. Consequently, the study cannot quantify the relative improvement produced by the tested crops compared with natural succession, unmanaged bare soil or background soil conditions. The observed vegetation cover, biomass production and visible stress responses therefore demonstrate the field feasibility of crop establishment on the investigated contaminated substrate, but they do not provide a controlled comparison against an unmanaged or reference condition. Future experimental phases should include unplanted plots, spontaneous vegetation controls and reference soils in order to better quantify the added value of active crop-based phytomanagement compared with passive revegetation or natural recovery [19,20].

A further limitation is that the Living Lab platform was established at one post-mining site only. Therefore, the results reflect the specific substrate, climatic and management conditions of the investigated Jiu Valley location and should not be generalised directly to all post-mining soils or mining regions. Future studies should include spatial replication across several post-mining sites with different substrate properties, contamination profiles and rehabilitation histories in order to test the broader applicability of the proposed phytomanagement approach [19,20].

Future research should therefore include extended monitoring of crop performance, seasonal biomass production, vegetation persistence and changes in soil physicochemical properties. Additional analyses should include bioavailable metal fractions, root and shoot metal partitioning, transfer factors, rhizosphere processes, soil oxygen or redox-related parameters, chlorophyll content, oxidative stress indicators, programmed cell death markers and potential changes in contaminant mobility over time [17,31]. Further studies should also investigate assisted phytomanagement options, including organic amendments, biochar, compost, microbial inoculants and other soil-improvement strategies, in order to enhance crop establishment, soil functionality and phytostabilisation efficiency under mining-affected field conditions [2,14,21,32,33]. For biomass reuse, future work should assess biomass quality, calorific properties, ash composition, digestate composition and contaminant fate during potential conversion or processing pathways [5,10,12,29,30].

Despite these limitations, the present field-based assessment provides useful practical evidence regarding the feasibility of establishing tolerant biomass crops on mining-affected soils. The results indicated that sorghum, and to a lesser extent maize, showed preliminary potential to support remediation-oriented phytomanagement strategies by increasing vegetation cover and biomass formation on contaminated post-mining surfaces. However, the actual contribution of these crops to reducing bare soil exposure, erosion-related contaminant dispersion and long-term ecological rehabilitation should be confirmed through future controlled comparisons including unplanted plots, spontaneous vegetation controls and reference soils [2,3,5].

## 5. Conclusions

This field-based Living Lab study assessed the preliminary feasibility of sorghum, maize and soybean for the phytomanagement of heavy metal-contaminated post-mining soil from the Jiu Valley, Romania. The results showed clear species-specific differences in establishment, vegetation cover, fresh aboveground biomass formation and aboveground metal occurrence. Total fresh biomass increased from 85 kg in Year I to 487 kg in Year III, indicating progressive vegetation development under the investigated field conditions. Sorghum showed the strongest preliminary phytomanagement potential, with the highest final biomass production, plot-level vegetation cover and suitability score. Maize showed intermediate feasibility, whereas soybean appeared more sensitive to the degraded substrate. Aboveground Cr, Cu, Ni, Zn and Pb occurrence confirmed that harvested biomass should be restricted to controlled non-food pathways only. These findings should not be interpreted as proof of complete phytostabilisation, contaminant removal or demonstrated reduction of erosion, runoff, dust dispersion or contaminant redistribution, because these processes were not directly measured. Future research should include dry biomass determination, root–shoot metal partitioning, bioavailable metal fractions, post-cultivation soil monitoring and metal-fate assessment during biomass conversion.

## Figures and Tables

**Table 1:** Scoring descriptors used for the semi-quantitative phytomanagement suitability assessment.

Score	Descriptor	Interpretation
1	Very low suitability	Poor establishment, severe stress symptoms, low vegetation development and limited contribution to surface stabilisation
2	Low suitability	Uneven establishment, evident stress symptoms, weak biomass development and reduced stabilisation potential
3	Moderate suitability	Acceptable establishment, moderate stress symptoms and limited to moderate contribution to vegetation cover and stabilisation
4	High suitability	Good establishment, limited stress symptoms, stable biomass development and clear contribution to soil cover
5	Very high suitability	Uniform establishment, high vigour, stable biomass development and strong potential for soil surface stabilisation

**Table 2:** Summary statistics of physicochemical properties and pseudo-total heavy metal concentrations across the nine composite soil samples collected before vegetation establishment.

Parameter	Unit	Mean Value	Range	Interpretation
pH	–	6.7	6.3–7.1	Slightly acidic to near-neutral conditions
Organic matter	%	2.4	1.8–3.1	Low organic matter content typical of degraded substrates
Chromium (Cr)	mg kg^−1^	118	82–146	Mining-related enrichment
Copper (Cu)	mg kg^−1^	74	51–92	Moderate enrichment
Nickel (Ni)	mg kg^−1^	63	41–79	Detectable contamination
Zinc (Zn)	mg kg^−1^	214	156–287	Highest concentration and variability among the analysed metals
Lead (Pb)	mg kg^−1^	96	64–121	Persistent contamination signal

Note: Metal concentrations are expressed as mg kg^−1^ dry weight and refer to pseudo-total concentrations obtained after microwave-assisted acid digestion. These values characterise the overall contamination status of the experimental substrate and do not directly indicate metal bioavailability.

**Table 3:** Regulatory interpretation of pseudo-total metal concentrations according to Romanian Order No. 756/1997.

Metal	Mean/Range in This Study (mg kg^−1^)	Normal Value (mg kg^−1^)	Alert Thresholds S/LS (mg kg^−1^)	Intervention Thresholds S/LS (mg kg^−1^)	Regulatory Interpretation
Cr	118/82–146	30	100/300	300/600	Above normal value; partly above sensitive-use alert threshold; below less-sensitive alert and intervention thresholds
Cu	74/51–92	20	100/250	200/500	Above normal value; below alert and intervention thresholds for both land-use categories
Ni	63/41–79	20	75/200	150/500	Above normal value; upper range slightly above sensitive-use alert threshold; below intervention thresholds
Zn	214/156–287	100	300/700	600/1500	Above normal value; below alert and intervention thresholds for both land-use categories
Pb	96/64–121	20	50/250	100/1000	Above sensitive-use alert threshold; upper range locally exceeds sensitive-use intervention threshold; below less-sensitive alert and intervention thresholds

Note: S: sensitive land use; LS: less sensitive land use. Thresholds are based on Romanian Order No. 756/1997 [26]. The comparison was used only for regulatory contextualisation of the contamination status. Measured values represent pseudo-total concentrations and should not be interpreted as direct indicators of metal bioavailability.

**Table 4:** Structured visual field assessment of crop establishment and vegetation development under mining-affected substrate conditions.

Field Indicator	*Sorghum bicolor*	*Zea mays*	*Glycine max*
Establishment uniformity	High; relatively uniform plot occupation	Moderate to high; good establishment, but slightly less uniform than sorghum	Moderate; less uniform establishment
Vegetation density	High	Moderate to high	Moderate to low
Canopy development	Dense and stable canopy development	Moderate to high canopy development	Less consistent canopy development
Visible stress symptoms	Low; limited chlorosis or growth reduction	Moderate; occasional chlorosis and reduced vigour	Moderate to high; more evident early-stage stress symptoms
Aboveground biomass formation	Comparatively high and stable	Moderate to high	Moderate to low
Surface stabilisation potential	High; strong contribution to soil cover	Moderate to high; good but less uniform soil cover	Moderate to low; weaker surface protection
Overall phytomanagement adaptability	High	Moderate to high	Moderate to low

Note: The assessment is based on repeated field observations and photographic records interpreted using predefined qualitative classes. Quantitative biomass, growth, vegetation cover and plant tissue metal data are presented separately in Table 5, Table 6 and Table 7.

**Table 5:** Multi-year species-level fresh aboveground biomass and growth indicators for the three tested crops.

Year	Fresh Biomass—Sorghum (kg)	Fresh Biomass— Maize (kg)	Fresh Biomass — Soybean (kg)	Total Fresh Biomass (kg)	Sorghum Height (cm)	Maize Height (cm)	Soybean Height (cm)	Sorghum Density (Plants m^−2^)	Maize Density (Plants m^−2^)	Soybean Density (Plants m^−2^)
Year I	46	13	26	85	75	60	8	14	16.5	11
Year II	125	78	60	263	80	75	10	17	18	14
Year III	230	126	131	487	130	125	12	20	19	17
Mean ± SD	133.7 ± 92.3	72.3 ± 56.7	72.3 ± 53.6	278.3 ± 201.3	95.0 ± 30.4	86.7 ± 34.0	10.0 ± 2.0	17.0 ± 3.0	17.8 ± 1.3	14.0 ± 3.0

Note: Year I, Year II and Year III refer to the first, second and third consecutive cultivation years of the Living Lab experiment, respectively. Fresh biomass values in this table refer to species-level harvested biomass for each cultivation year and are not directly comparable with the standardised plot-level assessment values reported in Table 6 unless normalised by harvested area.

**Table 6:** Plot-level fresh biomass recorded in the standardised final assessment area and vegetation cover in the Living Lab platform.

Plot	Species	Fresh Biomass (kg)	Assessed Area (m^2^)	Fresh Biomass (kg m^−2^)	Vegetation Cover (%)	Visible Stress	Interpretation
S1	Sorghum	10.20	64	0.159	55	Low	Good cover; moderate biomass
S2	Sorghum	10.24	64	0.160	75	Low	High cover; stable establishment
S3	Sorghum	25.56	64	0.399	86	Low	Highest biomass and cover
M1	Maize	1.50	64	0.023	25	Moderate	Low cover and biomass
M2	Maize	2.50	64	0.039	35	Moderate	Partial establishment
M3	Maize	9.00	64	0.141	60	Low-moderate	Good local performance
SO1	Soybean	4.80	64	0.075	30	Moderate	Lower early cover
SO2	Soybean	5.10	64	0.080	50	Moderate	Intermediate cover
SO3	Soybean	7.11	64	0.111	70	Low-moderate	Good cover, lower height
Mean ± SD	Sorghum	15.33 ± 8.86	64	0.240 ± 0.138	72.0 ± 15.7	–	Highest plot-level biomass and cover
Mean ± SD	Maize	4.33 ± 4.07	64	0.068 ± 0.064	40.0 ± 18.0	–	Lower mean cover at plot scale
Mean ± SD	Soybean	5.67 ± 1.26	64	0.089 ± 0.020	50.0 ± 20.0	–	Intermediate cover but lower growth vigour

Note: Plot-level values were used for descriptive statistics and exploratory non-parametric interpretation. Vegetation cover was estimated from five 1 m^2^ quadrats positioned systematically within the central 8 × 8 m assessment area of each plot. Fresh biomass values refer to the standardised 64 m^2^ biomass assessment area and should not be interpreted as biomass collected from the five quadrats used for vegetation cover estimation. The limited number of operational spatial replicates supports screening-level comparison, not definitive agronomic ranking.

**Table 7:** Concentrations of selected heavy metals in aboveground biomass of sorghum, maize and soybean collected at final harvest in the third cultivation year (2025 composite plant samples).

Species	Analysed Tissue	Cr (mg kg^−1^ dw)	Cu (mg kg^−1^ dw)	Ni (mg kg^−1^ dw)	Zn (mg kg^−1^ dw)	Pb (mg kg^−1^ dw)
*Sorghum bicolor*	Aboveground biomass	6.21	8.77	10.96	38.01	4.75
*Zea mays*	Aboveground biomass	7.75	10.64	12.92	47.11	6.46
*Glycine max*	Aboveground biomass	8.06	9.14	7.48	42.36	4.32

Note: Concentrations are expressed on a dry weight basis. Values correspond to species-level composite samples of aboveground biomass; roots were not analysed. Only Cr, Cu, Ni, Zn and Pb are reported, as these elements were common to both the soil and plant datasets and were used for the phytomanagement interpretation. Because plant tissue values correspond to species-level composite samples, they were not used for plot-level statistical comparison or for calculating total aboveground metal loads.

**Table 8:** Screening-level aboveground accumulation ratios (AR) calculated for the tested crops.

Species	Cr	Cu	Ni	Zn	Pb
*Sorghum bicolor*	0.053	0.119	0.174	0.178	0.049
*Zea mays*	0.066	0.144	0.205	0.220	0.067
*Glycine max*	0.068	0.124	0.119	0.198	0.045

Note: AR values were calculated as the ratio between metal concentration in aboveground plant biomass and the mean pseudo-total concentration of the same metal in soil. Values are dimensionless and should be interpreted only as screening-level indicators, not as true bioaccumulation factors based on plant-available metal fractions.

**Table 9:** Semi-quantitative phytomanagement suitability assessment of the investigated crops.

Evaluation Criterion	*Sorghum bicolor*	*Zea mays*	*Glycine max*
Vegetation establishment	5	4	3
Stress tolerance	5	4	3
Biomass stability	5	4	3
Contribution to ecological stabilisation	5	4	3
Potential for controlled non-food biomass use	4	3	2
Overall suitability score	24/25	19/25	14/25
Overall interpretation	High suitability	Moderate to high suitability	Moderate to limited suitability

Note: The potential for controlled non-food biomass use was scored conservatively using biomass production and aboveground plant metal concentrations. Root accumulation, ash composition, digestate quality and contaminant fate during conversion were not assessed in the present study.

**Table 10:** Field and quantitative evidence supporting the semi-quantitative phytomanagement suitability scores.

Evaluation Criterion	*Sorghum bicolor*	Score	*Zea mays*	Score	*Glycine max*	Score
Vegetation establishment	Uniform emergence and good plot occupation	5	Good emergence, but slightly less uniform than sorghum	4	Less uniform establishment and weaker plot occupation	3
Stress tolerance	Limited visible chlorosis or growth reduction	5	Occasional chlorosis and reduced vigour	4	More evident early-stage stress symptoms and reduced vigour	3
Biomass stability	Stable canopy development and dense vegetation cover during the growing season	5	Moderate to high aboveground development, but less uniform than sorghum	4	Lower and less stable aboveground development	3
Contribution to ecological stabilisation	Strong soil cover and clear contribution to surface protection	5	Good contribution to soil cover, but with lower uniformity	4	Moderate to low surface protection due to reduced vegetation density	3
Potential for controlled non-food biomass use	High biomass production, strongest cover and lowest Cr among the analysed aboveground samples; controlled non-food use only	4	Good biomass increase, but higher aboveground Cu, Ni, Zn and Pb; controlled non-food use only	3	Lower growth performance and highest Cr among the analysed aboveground samples; restricted non-food use only	2

Note: The scores integrate repeated field observations, plot-level vegetation indicators, biomass production and aboveground plant metal concentrations. They are intended to support comparative crop prioritisation for phytomanagement and should not be interpreted as definitive contaminant removal efficiencies or unrestricted biomass safety indicators.

## Data Availability

The raw data supporting the conclusions of this article will be made available by the authors on request.
